# Nutritional status and clinical evolution of the elderly in home enteral nutritional therapy: a retrospective cohort study*

**DOI:** 10.1590/1518-8345.2837.3198

**Published:** 2019-10-14

**Authors:** Caroline Soares Menezes, Renata Costa Fortes

**Affiliations:** 1 Fundação de Ensino e Pesquisa em Ciências da Saúde, Escola Superior de Ciências da Saúde, Brasília, DF, Brasil.; 2 Governo do Distrito Federal, Secretaria de Estado de Saúde do Distrito Federal, Brasília, DF, Brasil.

**Keywords:** Aged, Nutrition Therapy, Protein-Energy Malnutrition, Nutritional Status, Enteral Nutrition, Home Care Services, Idoso, Terapia Nutricional, Desnutrição Proteico-Calórica, Estado Nutricional, Nutrição Enteral, Serviços de Assistência Domiciliar, Anciano: Terapia Nutricional, Desnutrición Proteico-Calórica, Estado Nutricional, Nutrición Enteral, Servicios de Atención de Salud a Domicilio

## Abstract

**Objective:**

to evaluate the clinical and nutritional evolution of elderly patients receiving home enteral nutritional therapy.

**Method:**

retrospective cohort observational study. Data collection was performed through the analysis of clinical and nutritional records. The demographic, nutritional and clinical variables were analyzed. The sample consisted of elderly patients using home enteral nutritional therapy via the probe or the stomach. For the statistical analysis, the Statistical Package for the Social Sciences program was used, adopting the level of significance of 5%.

**Results:**

the sample was 218 participants, with a mean age of 76 ± 10.12 years, of which 54.1% were female. The main morbidity was the stroke sequelae. Malnutrition was the nutritional diagnosis and the overall subjective assessment, the main instrument of nutritional evaluation. The route of administration of the most prevalent diet was the nasoenteric/nasogastric tube, however, after one year of follow-up, gastrostomy became the main route. It was observed the predominance of general condition maintenance and the most prevalent clinical outcome was death.

**Conclusion:**

the majority of patients in home enteral nutrition therapy presented maintenance and / or improvement of clinical and nutritional status. Therefore, this therapy may contribute to a better clinical and nutritional evolution.

## Abstract (pt)

**Objetivo:**

avaliar a evolução clínica e nutricional de idosos que recebem terapia nutricional enteral domiciliar.

**Método:**

estudo observacional do tipo coorte retrospectiva. A coleta de dados foi realizada por meio de análise de prontuários dos registros clínicos e nutricionais. Foram analisadas as variáveis demográficas, nutricionais e clínicas. A amostra foi constituída por pacientes idosos em uso de terapia nutricional enteral domiciliar por via sonda ou estomia. Para a análise estatística, utilizou-se o programa *Statistical Package for the Social Sciences*, adotando-se o nível de significância de 5%.

**Resultados:**

a amostra foi de 218 participantes, com uma média de idade de 76±10,12 anos, sendo 54,1% do sexo feminino. A principal morbidade foi a sequela por acidente vascular encefálico. A desnutrição foi o diagnóstico nutricional e a avaliação subjetiva global, o principal instrumento de avaliação nutricional. A via de administração da dieta mais prevalente foi a sonda nasoentérica/nasogástrica, contudo, após um ano de acompanhamento, a gastrostomia passou a ser a principal via. Constatou-se o predomínio de manutenção do estado geral e o desfecho clínico mais prevalente foi o óbito.

**Conclusão:**

a maioria dos pacientes em terapia de nutrição enteral domiciliar apresentou manutenção e/ou melhora do quadro clínico e nutricional. Logo, essa terapia pode contribuir com uma melhor evolução clínica e nutricional.

## Abstract (es)

**Objetivo:**

evaluar la evolución clínica y nutricional de ancianos que reciben terapia nutricional enteral domiciliaria.

**Método:**

estudio observacional tipo cohorte retrospectiva. La recogida de datos fue realizada por medio de análisis de los registros clínicos y nutricionales. Fueron analizadas las variables demográficas, nutricionales y clínicas. La muestra estuvo constituida por pacientes ancianos que usaban terapia nutricional enteral domiciliaria por vía de sonda o gastrostomía. Para el análisis estadístico, se utilizó el programa *Statistical Package for the Social Sciences*, adoptando el nivel de significación de 5% Resultados: la muestra estuvo compuesta de 218 participantes con una media de edad de 76±10,12 años, siendo 54,1% del sexo femenino. La principal morbilidad fue la secuela por accidente vascular encefálico. La desnutrición fue el diagnóstico nutricional y la evaluación subjetiva global el principal instrumento de evaluación nutricional. La vía de administración de la dieta más prevalente fue la sonda nasoenteral/nasogástrica; sin embargo, después de un año de acompañamiento, la gastrostomía pasó a ser la principal vía. Se constató el predominio de manutención del estado general y el resultado clínico más prevalente fue la muerte.

**Conclusión:**

la mayoría de los pacientes en terapia de nutrición enteral domiciliaria presentó manutención y/o mejoría del cuadro clínico y nutricional. Luego, esa terapia puede contribuir con una mejor evolución clínica y nutricional.

## Introduction

The growth of the elderly population is a worldwide phenomenon and, in Brazil, the population is gradually aging^1^. With this demographic transition, there is a growing increase in the incidence of chronic non-communicable diseases, which has a major impact on health systems^1-2^. Thus, the number of elderly with home enteral nutritional therapy (HENT) has grown worldwide^3^.

HENT refers to nutritional assistance related to nutrient administration through home enteral nutrition^4^; promotes discharge and reintegration into the family nucleus. In addition, de-hospitalization stimulates the humanization of care, provides bed rotation, reduces iatrogenic risks and treatment costs^5^.

The Department of Health of the Federal District (SES-DF) has a program for the provision of special-purpose formulas - Home Enteral Nutrition Therapy Program (HENTP), regulated by Administrative Rule number 478, dated September 6, 2017. The program patients with indication of HENT by probe or ostomies and specific cases of oral supplementation^6^.

The elderly in HENT can enter home care with risk of malnutrition or installed malnutrition, but may also become malnourished during home care^4^. And, considering also the need to emphasize the importance of incentives to HENT programs within the scope of Unified Health System (UHS), the objective of this study was to evaluate the clinical and nutritional evolution of elderly individuals receiving HENT in the SES-DF HENTP.

## Method

It is an observational retrospective and analytical cohort study. It was approved by the Ethics and Research Committee on Human Subjects of the Health Science Teaching and Research Foundation (CEP / FEPECS) with the Certificate of Ethics Presentation Certificate number (CAAE) 57852616.6.0000.5553 and opinion number 1,656,435.

Data collection was performed at the Nutrition Management (GENUT) of SES-DF through a medical records analysis, for five months, from September 2016 to February 2017. GENUT is responsible for the HENTP and, through the Home Nutrition Central (HNUC), performs the acquisition and dispensation of the formulas; analyzes, controls and archives patient data and nutritional prescriptions; conducts audit visits to households; organizes meetings and training for professionals who provide service to the user; issues technical advice on formulas and participates in any flow of program documentation^6^.

The sample consisted of patients aged 60 years or older (definition of elderly by the Statute of the Elderly - Law 10.741, of October 1, 2003), using TNED by catheter (nasoenteric - SNE and nasogastric - SNG) or ostomy (gastrostomy - GTT and fasting-ostomy - JJT), enrolled in the HENT during the period from April 1, 2015 to September 30, 2015. Those whose essential data for this study were absent and / or illegible were excluded.

The data collection was performed through the clinical and nutritional records of the elderly enrolled, considering five moments: the evaluation of entry into the program and four subsequent reevaluations. The analyzed variables were: sex; age; regional service and residence; underlying disease; route of administration; anthropometry (height, weight and body mass index - BMI); nutritional diagnosis; gastrointestinal tract intercurrences (vomiting, diarrhea, constipation, flatulence, pain and abdominal distension); characteristics of the prescribed nutritional formula and clinical outcomes (presence of pressure lesions, readmissions and death).

At the SES-DF, patient care is performed at the regional health service according to the household, classification of the regional health services of the Federal District according to the Regionalization Master Plan - PDR-2013. It is divided in: Center-North (Asa North, Cruise and North Lake); Central-South (Asa Sul, Guará, Lago Sul, Candangolândia, Núcleo Bandeirante, Riacho Fundo I and II and Park Way); North (Planaltina, Sobradinho, Mestre D ‘Armas and Arapoanga); Leste (Paranoá and São Sebastião); West (Ceilândia and Brazlândia) and Southeast (Taguatinga, Samambaia and Recanto das Emas). Thus, the nutritional prescription of the elderly is done by SES-DF nutritionists, in each region, in addition to filling the data in a specific form, containing demographic, clinical and nutritional information. Every three months, patients should be reevaluated for adjustment / revalidation of the prescription or discharge of the program.

Statistical analysis was performed by the BMI Statistical Package for the Social Sciences (SPSS), version 20, for windows. The descriptive statistics were presented by the mean, standard deviation and frequencies. The Kolmogorov Smirnov test showed the normal distribution of the data and, thus, the descriptive analyzes and the comparison tests were performed by the ANOVA test. The correlation between the variables was verified by the Tukey HOC POST multiple comparisons test. The presence of statistical significance was determined according to the probability of p-value <0.05 and confidence interval (CI) of 95%.

## Results

According to GENUT data, from January 2018, 3020 patients were attended by HENTP from September 2016 to February 2017. Of these, 856 (28%) were elderly using HENT both by probe / stoma and oral routes. The sample of this study comprised 218 patients, which represents 25.5% of the total of the elderly assisted by the HENTP and 100% of the elderly with probe or ostomies that entered this program in the mentioned period.

The patients analyzed had a mean age of 76 ± 10.12 years and the female sex represented 54.1% (n = 118). The main regional service was the Center-South, comprising 26.1% (n = 57) of the services, however, the Southwest region, with 24.3% (n = 53), was the one that comprised the highest percentage of residence of patients (Table 1).

**Table 1:** Distribution of the elderly attended by the HENTP* by regional of service and regional of residence. Brasília, DF, Brazil, 2016-2017

Regional Administratives	Regional of Customer Service	Regional of Residence

	n (%)	n (%)
Mid-suth	57 (26.1%)	34 (15.6%)
Mid-north	20 (9.2%)	22 (10.1%)
West	26 (11.9%)	42 (19.3%)
Southwest	41 (18.8%)	53 (24.3%)
North	20 (9.2%)	25 (11.5%)
West	10 (4.6%)	15 (6.9%)
South	23 (10.6%)	27 (12.4%)
Agreed^†^	21 (9.6%)	

*HENTP = Home Enteral Nutritional Therapy Program; ^†^Agreed refer only to the regional service for including the hospitals of the network agreed to the Department of Health to register patients in the Home Enteral Nutrition Therapy Program. Hospital Universitário de Brasília, Hospital das Armed Forces and SARAH Network

The sample consisted of 218 patients who performed at least one evaluation in the program: 115 (52.8%) performed at least one reevaluation; 90 (41.3%) had at least two reassessments; 74 (34.9%) achieved at least three reevaluations and only 28% (n: 61) of the sample reached one year of follow-up, with four reevaluations. From the first to the last reevaluation, a 72% decrease (n = 157) in the sample.

Of the 115 patients who underwent a reevaluation, 37.3% (n = 41) presented readmissions, with an average hospitalization time of approximately 30 days (29.72 ± 3.38 days, maximum admission time of 150 days). When evaluating patients who completed one year of follow-up, a percentage of approximately 38% (n = 23) of readmissions were observed, with a mean time of hospitalization of 21 days (± 2.36), 122 days and the minimum of one day.

The main morbidity identified was sequelae due to stroke, representing 31.2% (n = 68) of the diagnoses presented, following the dementia diseases, with approximately 26% (n = 56) of the cases. The cancer represented 22.5% (n = 49), 13.8% (n = 30) in the gastrointestinal tract and 8.7% (n = 19) of other types; 45 (20.6%) patients had other diagnoses.

In relation to anthropometry, a mean height (meters) of 1.60 ± 0.09, weight (kg) of 54 ± 11.64 and BMI (kg / m^2^) of 21.04 ± 4.24. When analyzing the method of weight gain, it was verified that the most used technique was the estimation, with 75.6% (n = 68) of the general sample, followed by the measurement, with 14.4% (n = 13) and by weight, with 10% (n = 9). In the patients who completed one year of follow-up, weight estimation continued to be the most used technique (71.4%, n = 30), followed by the 19% (n = 8) and 9.5% (n = 4). However, there was no report of the technique of weight gain in 58.7% (n = 128) of the general sample and 31.1% (n = 19) of the sample that completed one year of follow-up.

Regarding the technique of obtaining stature, it was found that the most used was the estimate, with 73.4% (n = 58) of the general sample, followed by the stated height, with 16.5% (n = 13) and by the measurement, with 10.1% (n = 8). In the patients who completed one year of follow-up, the estimation remained the most used technique (n = 28, 76.9%), followed by the stated height, with 19.2% (n = 7) and by the 3, 8% (n = 1). However, 63.8% (n = 139) of the general sample and 55.7% (n = 25), who completed one year of follow-up, had missing data regarding the method of obtaining height.

The Global Subjective Assessment (GSA) was the most used nutritional assessment (34.3%, n = 47), followed by the Mini Nutritional Assessment - MNA (32.1%, n = 44); however, there was no report of the type of evaluation in 81 (37.2%) cases. On completing the four reassessments, it was observed that the most applied method was MNA in about 52.4% of the patients (Table 2).

**Table 2:** Distribution of the elderly attended by the HENTP* by type of nutritional evaluation per analyzed period. Brasília, DF, Brazil, 2016-2017

Nutrition assessment	Input assessment^†^	First reevaluation^‡^	Second revaluation^§^	Third revaluation^||^	Fourth reassessment^¶^

	n (%)	n (%)	n (%)	n (%)	n (%)
GSA**	47 (34.3%)	12 (17.1%)	6 (10.5%)	5 (11.4%)	2 (4.7%)
CompletE^††^	9 (6.6%)	3 (4.3%)	2 (3.5%)	1 (2.3%)	0 (0.0%)
MNA^‡‡^	44 (32.1%)	35 (50.0%)	32 (56.1%)	22 (50.0%)	22 (52.4%)
NRS - 2002^§§^	5 (3.6%)	1 (1.4%)	1 (1.8%)	1 (2.3%)	0 (0.0%)
Other^||||^	32 (23.4%)	19 (27.2)	16 (28.1%)	15 (34.0%)	18 (42.9%)
Total informed	137 (100%)	70 (100%)	57 (100%)	44 (100%)	42 (100%)
Informed^¶¶^	137 (62.8%)	70 (60.9%)	57 (65.6%)	44 (59.5%)	42 (68.9%)
Absent***	81 (37.2%)	45 (39.1%)	31 (34.4%)	30 (40.5)	19 (31.1%)
Total	218 (100%)	115 (100%)	90 (100%)	74 (100%)	61 (100%)

* HENTP = Home Enteral Nutritional Therapy Program; ^†^Intake evaluation refers to the first nutritional evaluation performed at the time of patient’s entry into the Home Enteral Nutritional Therapy Program; ^‡^First reevaluation is the nutritional reevaluation carried out three months after entering the program; ^§^Second reassessment is the nutritional reassessment performed six months after entry into the program and three months after the first nutritional reevaluation; ^||^The third reassessment is the nutritional reassessment performed nine months after entry into the program and three months after the second nutritional reassessment; ^¶^Fourth reassessment is the nutritional reassessment performed 12 months after entry into the program and three months after the third nutritional reassessment; **ASG = Global Subjective Assessment; ^††^Full refers to objective nutritional assessment; ^‡‡^MAN = Mini Nutritional Assessment; ^§§^NRS-2002 = Nutritional Risk Screening-2002; ^||||^Another refers to all other types of nutritional assessment described in patient charts; ^¶^Informed refers to the number of patients who presented information about the type of nutritional evaluation performed in the care; ***Absentee refers to the number of patients who did not present information of the type of nutritional evaluation performed in the care

The most prevalent initial nutritional diagnosis was malnutrition, representing 65.1% (n = 142) of the cases, and in the group that completed the four reevaluations, this prevalence reduced, in absolute numbers, to 47.5% (n = 29), according to Table 3.

**Table 3:** Distribution of the elderly attended by the HENTP* by nutritional diagnosis for the period analyzed. Brasília, DF, Brazil, 2016-2017

Nutrition assessment	Input assessment^†^	First reevaluation^‡^	Second revaluation^§^	Third revaluation^||^	Fourth reassessment^¶^

	n (%)	n (%)	n (%)	n (%)	n (%)
Malnutrition	142 (65.2%)	77 (67.0%)	51 (56.7%)	39 (52.7%)	29 (47.6%)
Eutrophy	65 (29.8%)	31 (26.9%)	32 (35.6%)	29 (39.2%)	28 (45.9%)
Overweight	7 (3.2%)	3 (2.6%)	4 (4.5%)	4 (5.4%)	1 (1.6%)
Obesity	4 (1.8%)	4 (3.5%)	3 (3.3%)	2 (2.7%)	3 (4.9%)
Total	218 (100%)	115 (100%)	90 (100%)	74 (100%)	61 (100%)

*HENTP = Home Enteral Nutritional Therapy Program; ^†^Intake evaluation refers to the first nutritional evaluation performed at the time of patient’s entry into the Home Enteral Nutritional Therapy Program; ^‡^First reevaluation is the nutritional reevaluation carried out three months after entering the program;^§^Second reassessment is the nutritional reassessment performed six months after entry into the program and three months after the first nutritional reevaluation; ^||^The third reassessment is the nutritional reassessment performed nine months after entry into the program and three months after the second nutritional reassessment; ^¶^Fourth reassessment is the nutritional reassessment performed 12 months after entry into the program and three months after the third nutritional reassessment

The route of administration of the most prevalent diet was NEC / NGC (62.4%, n = 136), followed by GTT (36.2%, n = 79). In the patients who reached one year of follow-up, GTT became the main route of administration (75.1%, n = 45), according to Table 4.

**Table 4:** Distribution of the elderly attended by the HENTP* through diet administration per analyzed period. Brasília, DF, Brazil, 2016-2017

Nutrition assessment	Input assessment^†^	First reevaluation^‡^	Second revaluation^§^	Third revaluation^||^	Fourth reassessment^¶^

	n (%)	n (%)	n (%)	n (%)	n (%)
GTT**	79 (36,2%)	68 (59,2%)	63 (70,0%)	54 (72,9%)	45 (75,0%)
NEC^††^/NGC^‡‡^	136 (62,4%)	43 (37,4%)	25 (27,8%)	18 (24,3%)	13 (21,6%)
FO^§§^	3 (1,4%)	2 (1,7%)	2 (2,2%)	1 (1,4%)	1 (1,7%)
OR^||||^	0 (0,0%)	2 (1,7%)	0 (0,0%)	1 (1,4%)	1 (1,7%)
Total	218 (100%)	115 (100%)	90 (100%)	74 (100%)	61 (100%)

* HENTP = Home Enteral Nutritional Therapy Program; ^†^Intake evaluation refers to the first nutritional evaluation performed at the time of patient’s entry into the Home Enteral Nutritional Therapy Program; ^‡^First reevaluation is the nutritional reevaluation carried out three months after entering the program; §Second reassessment is the nutritional reassessment performed six months after entry into the program and three months after the first nutritional reevaluation; Third reevaluation is the nutritional reevaluation performed nine months after entry into the program and three months after the second nutritional reevaluation; ^¶^Fourth reassessment is the nutritional reassessment performed 12 months after entry into the program and three months after the third nutritional reassessment; **GTT = gastrostomy; ^††^NEC = nasoenteric catheter; ^‡‡^NGC = nasogastric catheter; ^§§^FO = fasting-ostomy; ^||||^OR = oral route

Regarding the clinical evolution, it was observed that 50.9% (n = 55) of the patients who performed at least one reassessment had a maintenance of the condition, 21.3 % (n = 23), improvement and 27.8% (n = 30) had, as a result, clinical worsening. When only the patients who performed the four reevaluations were analyzed, the predominance of general state maintenance (59.3%, n = 32), followed by clinical improvement (24.1%, n = 13) was observed, as shown in Table 5.

**Table 5:** Distribution of the elderly attended by the HENTP* by clinical evolution, for the analyzed period, disregarding the entrance evaluation. Brasília, DF, Brazil, 2016-2017

Nutrition assessment	Input assessment^†^	First reevaluation^‡^	Second revaluation^§^	Third revaluation^||^

	n (%)	n (%)	n (%)	n (%)
Maintenance	55 (50.9%)	45 (54.2%)	37 (54.4%)	32 (59.2%)
Improved	23 (21.3%)	23 (27.7%)	18 (26.5%)	13 (24.1%)
Worsens	30 (27.8%)	15 (18.1%)	13 (19.1%)	9 (16.7%)
Total informed	108 (100%)	83 (100%)	68 (100%)	54 (100%)
Informed^¶^	108 (93.9%)	83 (92.2%)	68 (91.9%)	54 (88.5%)
Absent**	7 (6.1%)	7 (7.8%)	6 (8.1)	7 (11.5%)
Total sample	115 (100%)	90 (100%)	74 (100%)	61 (100%)

*HENTP = Home Enteral Nutritional Therapy Program; ^†^First reevaluation is the nutritional reevaluation carried out three months after entering the program; ^‡^Second reassessment is the nutritional reassessment conducted six months after entry into the program and three months after the first nutritional reassessment; ^§^The third reassessment is the nutritional reassessment performed nine months after entry into the program and three months after the second nutritional reassessment; Fourth reevaluation is the nutritional reevaluation performed 12 months after entry into the program and three months after the third nutritional reevaluation; ^¶^Reported refers to the number of patients who presented information about the type of nutritional evaluation performed in the care; **Absentee refers to the number of patients who did not present information regarding the type of nutritional evaluation performed in the care

Regarding pressure ulcers (PU), there were only records of PU and its characteristics in 31 (14.2%) patients at the entrance evaluation and 17 (27.9%) patients with four nutritional reassessments. Of these, 80.6% (n = 25) had open PU, a predominance that was maintained in those who received four nutritional reassessments (52.9%, n = 9). In these patients, 5.9% (n = 1) presented PU in the healing phase, 17.6% (n = 3) had it healed and 23.5% (n = 4) did not present it.

In relation to GIT, only the absence of intercurrences was recorded in 32 (14.7%) patients at the entrance evaluation; the data of the remaining patients were absent (85.3%, n = 186). Similar data were observed in those who completed the four reassessments: 39 (63.9%) patients had no intercurrences of GIT; one (1.63%) had abdominal distension and 34.4% (n = 21) of the data were absent. Concerning intestinal function, approximately 88% (n = 191) of the patients of the entrance were with the data absent; (n = 16) reported regular function, 33.3% (n = 9), constipation and 7.4% (n = 2), and diarrhea. In patients who completed one year of follow-up, data were absent in 49.2% (n = 30) of the sample and a higher prevalence of regular bowel function (90.3%, n = 28) followed by diarrhea (6.5%, n = 2) and by constipation (3.2%, n = 1).

Regarding the clinical outcome, there was no information on the outcome in 27.1% (n = 59); however, of the 72.9% (n = 159) who had information, 25.8% (n = 41) continued in the program, 39% (n = 62) died and 35.2% (n = 56) were disqualified.

It was observed that 95.4% (n = 208) of the patients at the entrance evaluation and 98.3% (n = 60) of those who had four reassessments used the nutritionally complete polymer formula indicated for feeding via probes or ostomies, sucrose, lactose and gluten, plus fiber, with an energy density of 1.0 to 1.2 kcal / mL at the standard dilution and a protein content of 14 to 17% of the total caloric value. In the entrance evaluation, 3.2% (n = 7) received the indicated formula for disabsortive syndromes, based on oligomeric and / or monomeric protein, lactose-free and sucrose, with an energy density of 1.0 to 1.2 kcal / mL at the standard dilution and protein content of 13% to 20%. Only two (0.9%) patients, at the entrance evaluation, and one (1.6%), who had four reevaluations, used the descriptive index indicated for chronic kidney disease in dialysis treatment with polymeric protein, free of sucrose, lactose and gluten, with a caloric density of 1.5 to 2.0 kcal / mL and a protein content of 14 to 20% of the total caloric value.

The use of supplementation in enteral nutrition was performed by 11.9% (n = 26) and 9.8% (n = 6) patients, respectively in the one-year entry and reevaluation evaluation. Of these, 92.3% (n = 24) at the entrance and 83.3% (n = 5) at the one-year follow-up evaluation used the product indicated for the supplementation of patients with PU or congenital epidermolysis bullosa, and may contain other nutrients that assist in the healing of wounds, with or without sucrose, with or without fiber, with an energy density of 1,0 kcal / mL and a protein content of 20% or more of the total energy value.

Regarding the modules, 15.5% (n = 34) of the patients at the entrance were used, with casein being the most frequently used module (67.6%, n = 23), followed by the glutamine module (11.8%, n = 4). Others included fibers to regulate gastrointestinal transit (17.6%, n = 6), divided into soluble fiber (8.8%, n = 3) and insoluble and soluble (8.8%, n = 3), followed by the medium chain triglyceride module (MCT) (2.9%, n = 1). In the patients who completed the four re-evaluations, 26.2% (n = 16) use of modules was observed, with casein remaining the most prevalent module (56.3%, n = 9), followed (n = 3), soluble fiber modulus (12.5%, n = 2), glutamine (6.3%, n = 1) and long chain triglycerides (LCT) (6.3%, n = 1).

The following averages were considered: a height of 1.60 ± 0.09 meters, considering the anthropometric data of the sample; weight of 53.66 ± 11.64 kg and BMI of 21.0 ± 4.24 kg / m^2^. In the first reevaluation, the mean height was 1.60 ± 0.08 meters, weight of 51.94 ± 11.40 kg and BMI of 20.38 ± 4.27 kg / m^2^. In the second reevaluation, the mean height was 1.59 ± 0.08 meters, the mean weight was 53.47 ± 10.46 kg and the BMI was 21.17 ± 4.13 kg / m^2^. In the third re-evaluation, the averages found were 1.58 ± 0.08 meters in height, weight of 53.55 ± 10.10 kg and BMI of 21.38 ± 3.68 kg / m^2^. Finally, the fourth reevaluation found a mean height of 1.58 ± 0.07 meters, weight of 53.64 ± 9.99 kg and BMI of 21.46 ± 3.45 kg / m^2^. There were no statistically significant differences between age, weight, height and BMI in the different periods (p> 0.05).

## Discussion

The prevalence of elderly individuals aged 70 or over, as found in the sample studied, is compatible with the literature in relation to patients assisted in home care^5,7-11^. This fact can be explained by the process of demographic transition associated with the increase of disability levels, according to the rise of chronic diseases in aging^1^.

Similar to other studies on home care^7-9,11-12^, there was a predominance of females. This fact is justified by the higher mortality in the male population due to biological factors and / or unequal exposure to health risk factors. When analyzing the Federal District, there is also a greater expectation of life among women^1^.

Regarding the distribution by regional of attendance, it was observed a greater concentration of the attendance in the Central-South region, which is justified by the location of one of the largest hospitals in the Federal District. In relation to the regional of residence, the Southwest was the most prevalent; it has expanded and the satellite city of Taguatinga is among the highest percentage of elderly, according to the District Survey by Household Sample of the Federal District (PDAD/DF)^13^.

A decrease of 72% of the sample was observed between the entry in the program and the last re-evaluation. The main reason for leaving the HENTP was death, which can be justified by the profile of chronic diseases of patients associated with various sequelae and comorbidities, corroborating the data found in other studies^7,9-10^.

35.2% of the patients were discharged, and this discharge from the program can occur for several reasons, including the fact that the patient no longer has the criteria to continue in the HENTP, has evolved the oral diet, has not withdrawn the formula provided by program for more than six months and have moved from DF^6^. Discharge may be a positive outcome of the program, since improving the clinical condition may lead to non-inclusion in the criteria for home care.

Regarding the percentage of re-hospitalization, results lower than those found in this study were obtained from a survey of a home care service in the Northeast^7^ and in a study on Home Hospitalization Service in Southern Brazil^14^ in which 24% and 26.3% of the patients, respectively, presented hospital readmissions during home care. However, there is no standardized reference value to consider a high readmission rate and there is a lack of HENTP studies in Brazil^15^, which makes it difficult to judge the percentage found in the study. It should be pointed out that re-hospitalization is an indicator of quality of care that can be used to measure the resolution of home care^7^ *.*

The literature demonstrates a high prevalence of neurological disorders as the main clinical cause that leads to the use of HENT, with stroke being the most common diagnosis^7-8,10,14,16-19^. The study conducted in the Federal District with patients from the HENTP, in 2005, found a prevalence of 42.6% in the elderly with stroke^8^. In a survey carried out in a home care service in Maceió - Alagoas, the percentage of patients with stroke was 35.2%^7^, corroborating this study. These results corroborate the Spanish study that found a higher prevalence of neurological disorders^10,12^.

The measurement of weight and height of patients in HENT is not always possible due to the physical limitations of the patients. The estimation was the method most used in the study to obtain these parameters, the same technique used by two studies evaluated in a literature review^19^. Weight estimation may be by visual BMI or validated formula of weight suggestion, such as using the arm circumference measure^20^. Height can be estimated by the formula validated in 1985, using the measure of knee height^21^. From the data collected, it was not possible to know the type of method used in these anthropometric estimates.

The mean BMI in this study was 21.0 ± 4.24 kg / m^2^, which shows a tendency to malnutrition. Due to the physiological limitations imposed by the aging process, a BMI <22 kg / m^2^ is considered as a cutoff point for the classification of malnutrition in the elderly^22^. Even considering the limitations inherent to the use of BMI, it is emphasized that it is an indicator widely used in clinical and epidemiological studies^23^.

GSA and MNA were the most used subjective and consolidated nutritional assessments in this study. GSA was originally designed for the evaluation of surgical patients and was subsequently used and adapted to the different clinical situations, and MNA was developed and validated especially for the elderly. Both methods have good sensitivity and specificity, being considered adequate for the nutritional evaluation of the elderly and determination of the high risk for malnutrition and / or malnutrition already installed^23-25^.

A high prevalence of malnutrition (65.1%) was observed in the admission of HENTP patients, which is consistent with the literature, which reports that the elderly in HENT can already enter home care in the presence of risk of malnutrition or installed malnutrition^4^. These results are also plausible with other studies from Brazil and the world that found a high prevalence of malnutrition in patients admitted to home care^5,8,12^.

Malnutrition in the elderly is a public health problem due to physiological, nutritional, psychological and social factors. Weight loss in the elderly is often associated with sarcopenia (loss of muscle mass, strength and performance), which influences functional status and thus quality of life^1-2,26-27^. There is a relationship between the nutritional diagnosis and the severity of the disease^2,19^, and malnutrition has direct correlations with clinical complications such as mortality rate, pressure lesions and the number of readmissions^5,27^.

Regarding the route of administration, this study found a greater predominance of NEC / NGC, in agreement with the literature review conducted in 2014^19^. The study on home care in Maceió also found, in patients assisted by the public service, a higher prevalence of NEC / NGC^7^. In 2009, a study conducted in the Federal District also found a higher prevalence of NEC / NGC in HENTP patients. The authors justified that the high cost associated with the TWG in relation to the NEC / NGC access may have implications for the lower prevalence of this technique in the UHS^8^.

The “gold” standard for probe access is percutaneous endoscopic gastrostomy and its use is recommended when the tube feeding time is longer than two or three weeks, considering the lower risk of complications and higher quality of life^17-18,28^. In this study, there was an increase in GTT percentages (75%) in patients who reached one year of follow-up, a trend that was also observed in the literature^10^.

Regarding the clinical evolution, the maintenance of the general condition of the patients was predominant. This data is formed from a subjective evaluation of the nutritionist who is attending the patient and the patient caregiver’s report. The caregiver follows the evolution of the patient and becomes an important component of the care team ^29-30^. The maintenance of the general state is a good indicator of home care, since the worsening of the clinical picture, through complications, comorbidities and additional sequelae, is already a progress.

The PU analysis, in this study, presented an information bias, since the nutritional form standardized by the HENTP did not have a specific filling field on this lesion. Thus, the attending nutritionists, in most cases, only reported the presence of PU to justify the prescription of the specific product for healing. However, results similar to those found in this study were observed in the literature^7,31^, with PU prevalence around 20%. It should be mentioned that, in Ordinance number 478, effective as of 2017, information on the presence of PU in the nutritional form.

Nutritional intervention is essential and must be considered in the treatment of PU. The prescription of formulas with higher protein and immunomodulatory nutrients has been recommended because it interferes positively in the healing process^32^. As was observed in HENTP of DF, the most commonly used probe supplement was PU indicated, being normo-caloric, hyper-proteic and rich in arginine, besides the protein module being the most used, which characterizes the prescription of hyper-proteic diets.

The most common complication of GIT was intestinal constipation, a result similar to that found in other HENT studies in the literature^5,11^. On the other hand, an investigation that analyzed the challenges of HENT worldwide found diarrhea as the most prevalent complication^3^, the same one reported by the study carried out in Belo Horizonte - Minas Gerais^9^.

The most prescribed formula was polymeric, normocaloric, normolipidic and normoproteic plus fibers, in agreement with other researches^18,33^. Prescription should always consider the clinical condition, nutritional status, access route, and expected results of HENT.

No difference was found between the evaluations, which may demonstrate the benefits of HENT in not allowing the elderly to worsen their nutritional status. It is well elucidated that HENT is capable of guaranteeing nutritional needs, assisting in the recovery of nutritional status and providing tissue regeneration.

In spite of the results found in this study, the lack of data in the medical records is an important limitation, which justifies the need for greater awareness among the professionals that attend the elderly in the home, on the need for complete information, mainly aiming at patient safety, besides allowing future researches capable of subsidizing UHS actions.

However, it should be noted that the SES-DF HENTP highlights the importance of HENT and the advances in public policies aimed at the elderly population, as well as being a strategy for the de-hospitalization and humanization of UHS care.

## Conclusion

It was identified that the majority of patients in home enteral nutrition therapy presented maintenance and / or improvement of clinical and nutritional status. Through the clinical evolution, it was verified that the elderly patient in HENT has fewer numbers of re-hospitalizations and maintenance of the general condition. And, regarding the nutritional evolution, it was observed that HENT was able to avoid worsening nutritional status. These results indicate that the SES-DF HENTP is essential for the clinical and nutritional evolution of the patients assisted by it, besides representing a satisfactory strategy for the de-hospitalization and humanization of UHS care.
